# Exploratory genome-wide analyses of cortical inhibition, facilitation, and plasticity in late-life depression

**DOI:** 10.1038/s41398-023-02532-0

**Published:** 2023-06-30

**Authors:** Rafae A. Wathra, Xiaoyu Men, Samar S. M. Elsheikh, Victoria S. Marshe, Tarek K. Rajji, Jennifer I. Lissemore, Benoit H. Mulsant, Jordan F. Karp, Charles F. Reynolds, Eric J. Lenze, Zafiris J. Daskalakis, Daniel J. Müller, Daniel M. Blumberger

**Affiliations:** 1 Temerty Centre for Therapeutic Brain Intervention, Centre for Addiction and Mental Health, Toronto, Ontario M6J 1H4 Canada; 2 Department of Psychiatry, Temerty Faculty of Medicine, University of Toronto, Toronto, Ontario M5T 1R8 Canada; 3 Campbell Family Mental Health Research Institute, Centre for Addiction and Mental Health, Toronto, Ontario M5T 1R8 Canada; 4 Toronto Dementia Research Alliance, University of Toronto, Toronto, Ontario Canada; 5 Department of Psychiatry and Behavioral Sciences, Stanford University Medical Center, Stanford, CA USA; 6 Department of Psychiatry, University of Arizona College of Medicine, Tucson, AZ USA; 7 Department of Psychiatry, University of Pittsburgh School of Medicine, Pittsburgh, PA USA; 8 Department of Psychiatry, Washington University School of Medicine, St Louis, MO USA; 9 Department of Psychiatry, University of California San Diego, San Diego, CA USA

**Keywords:** Depression, Clinical genetics

## Abstract

Late-life depression (LLD) is a heterogenous mood disorder influenced by genetic factors. Cortical physiological processes such as cortical inhibition, facilitation, and plasticity may be markers of illness that are more strongly associated with genetic factors than the clinical phenotype. Thus, exploring the relationship between genetic factors and these physiological processes may help to characterize the biological mechanisms underlying LLD and improve diagnosis and treatment selection. Transcranial magnetic stimulation (TMS) combined with electromyography was used to measure short interval intracortical inhibition (SICI), cortical silent period (CSP), intracortical facilitation (ICF), and paired associative stimulation (PAS) in 79 participants with LLD. We used exploratory genome-wide association and gene-based analyses to assess for genetic correlations of these TMS measures. *MARK4* (which encodes microtubule affinity-regulating kinase 4) and *PPP1R37* (which encodes protein phosphatase 1 regulatory subunit 37) showed genome-wide significant association with SICI. *EGFLAM* (which encodes EGF-like fibronectin type III and laminin G domain) showed genome-wide significant association with CSP. No genes met genome-wide significant association with ICF or PAS. We observed genetic influences on cortical inhibition in older adults with LLD. Replication with larger sample sizes, exploration of clinical phenotype subgroups, and functional analysis of relevant genotypes is warranted to better characterize genetic influences on cortical physiology in LLD. This work is needed to determine whether cortical inhibition may serve as a biomarker to improve diagnostic precision and guide treatment selection in LLD.

## Introduction

Depression is a multifactorial disease influenced by both genetic and non-genetic factors [1]. In late-life, depression affects approximately 15% of older adults living in the community [2]. Aging and depression are both associated with changes in cortical physiology, such as cortical inhibition, excitation, and plasticity [3]. Exploring the relationship between genetic factors and cortical physiological processes could improve our understanding of biological mechanisms underlying late-life depression (LLD), leading to improved diagnosis and selection of treatment (i.e., biomarker-informed precision psychiatry).

Twin studies suggest that 16–55% of the variance in depressive symptoms in older adults may be due to genetic influences, and heritability may be greater with increased age [4, 5]. However, there has been limited success in identifying causal genetic loci for LLD [6]. In a meta-analysis, polymorphisms in apolipoprotein E (*APOE*), brain-derived neurotrophic factor (*BDNF*), and serotonin transporter (*SLC6A4*) genes were associated with LLD [6]. Genome-wide association studies (GWAS) of LLD have identified gene variants associated with cognitive decline and antidepressant response [7, 8]. However, environmental, epigenetic, and polygenic interactions make gene-depression signals difficult to detect and interpret [9].

Neural plasticity and cortical physiology may be potential pathways by which genes influence LLD. Transcranial magnetic stimulation (TMS) can be used to assess various neurophysiological measures. Short interval intracortical inhibition (SICI) measures the suppression of motor-evoked potential (MEP) amplitude when a suprathreshold TMS pulse is pre-conditioned by 1–6 ms with a subthreshold TMS pulse, which is indicative of GABA_A_ inhibitory activity [10]. The cortical silent period (CSP) measures the duration of electromyography suppression when a suprathreshold TMS pulse is given during a sustained muscle contraction, which is indicative of GABA_B_ inhibitory activity [11]. The resting motor threshold (RMT) is a general measure of neuronal excitability, which is influenced by ion channel conductivity [12]. Intracortical facilitation (ICF) measures relative MEP amplitude when a subthreshold TMS pulse precedes a suprathreshold TMS pulse by 10–15 ms, which is indicative of N-methyl-D-aspartate (NMDA) receptor-related excitatory activity [12]. Paired associative stimulation (PAS) refers to repeated median nerve stimulation preceding TMS stimulation and subsequent measurement of MEPs in a target muscle [13]. An interstimulus interval of approximately 25 ms causes an excitatory effect, and changes in subsequent MEP measurements indicate long-term potentiation (LTP) like cortical plasticity [13].

The interaction among depression, aging, and genetics in the pathophysiology of LLD remain unclear. Depression and advancing age have both been related to changes in cortical physiology. Attenuation in GABA_A_ receptor-mediated cortical inhibition has been demonstrated in patients with LLD, healthy older adults, and younger adults with depression [3]. Decreases in cortical excitability and impaired LTP-like plasticity have been shown in healthy older adults and patients with major depressive disorder (MDD) [14–16]. Existing GWAS and candidate gene studies have assessed genetic correlations of various proxy phenotypes of aging, including longevity, age-associated diseases, and physiological characteristics, such as muscle strength and cognitive function [17]. Several genes have been associated with aging, including *APOE*, *GRP78*, and *FOXO3A* [17]. Aging-related genes may influence measures of cortical physiology, as changes in cortical physiology have been observed in healthy older adults [14–16].

Few existing studies have assessed the association of genetic polymorphisms with cortical physiological processes measured by TMS. The *BDNF* gene encodes a protein that has been implicated in neuronal survival, neuroplasticity, and synaptogenesis [18]. Substitution of valine to methionine at codon 66 (Val66Met) is the most common and well-researched single nucleotide polymorphism (SNP) of *BDNF* [18]. Multiple studies suggest that there are no differences in SICI, CSP, ICF, or RMT dependent on the Val66Met SNP in healthy participants, though this has not been explored in LLD [19–22]. A few studies report that an increase in post-PAS MEP amplitudes is diminished in those with the Val66Met SNP [23–25]. Even fewer studies have assessed the impact of other SNPs on TMS indices of cortical physiology, and none that are specific to LLD [26–29].

Thus, we conducted the first exploratory GWAS of TMS cortical physiological processes in LLD. Based on the literature reviewed above, we hypothesized that *BDNF* polymorphisms would be associated with cortical physiology. Second, based on extensive literature on the relationship between neurotransmitters and depression [1, 30], we hypothesized that variations in genes encoding for serotonin [31, 32], norepinephrine [33], dopamine [34, 35], GABA [36, 37], and glutamate receptors and transporters [36, 37] would be associated with cortical physiology changes in LLD.

## Methods

### Participants

Participants were recruited from the Toronto site of two multi-center clinical trials (ClinicalTrials.gov Identifiers: NCT00892047 and NCT02263248). Participants completed TMS and genetic assessment prior to receiving any treatment intervention in the trials. Informed consent was obtained from all subjects. As described in details previously [38, 39], the main inclusion criteria were: age ≥ 50 (although most participants were 60 years and older), current diagnosis of MDD as per the Diagnostic and Statistical Manual of Mental Disorders, Fourth Edition, and Montgomery-Åsberg Depression Rating Scale (MADRS) [40] score ≥15. Exclusion criteria were a diagnosis of dementia; Mini-Mental State Examination (MMSE) score ≤21; a diagnosis of bipolar disorder or a psychotic disorder; substance misuse; an unstable medical condition; and anticonvulsant use. The sample for this analysis comprised 79 participants whose data (clinical, TMS neurophysiology, and genetic) passed quality control.

### TMS and electromyography measures

All TMS measures were conducted in accordance with international consensus guidelines [41]. TMS was delivered to the left motor cortex through a figure-of-eight coil (70 mm loop diameter) using two Magstim 200 stimulators (Magstim, Whitland, UK) connected by a Bistim module. Participants were asked to remain relaxed with their eyes open. Disposable 9 mm electrodes were attached to the abductor pollicis brevis muscle (active electrode) and the interphalangeal joint of the thumb (reference electrode) to measure surface electromyography (EMG). A ground electrode was positioned on the upper forearm. The figure-of-eight coil was held tangentially to the scalp over the left motor cortex “hot spot” to evoke maximum MEP strength, with the handle pointing 45 degrees from the midline to induce a posterior-to-anterior current flow. This position was marked on the scalp to ensure consistent coil placement.

RMT was defined as the lowest TMS intensity that elicited an MEP ≥ 50 μV in at least 5 of 10 consecutive trials. When assessing SICI, ICF, and PAS, a suprathreshold TMS test pulse was defined as the TMS intensity that evoked on average a ~1 mV peak-to-peak MEP amplitude. SICI and ICF were measured using paired-pulse TMS with a subthreshold conditioning stimulus (80% of RMT) followed by a test pulse with an interstimulus interval (ISI) of 2 ms (SICI) or 10 ms (ICF). Participants underwent 12 trials of test pulse alone (to determine the average unconditioned MEP amplitude), 12 trials of test pulse 2 ms after the conditioning pulse, and 12 trials of test pulse 10 ms after the conditioning pulse. SICI and ICF ratios were calculated as the average respective conditioned MEP amplitudes divided by the average unconditioned MEP amplitudes.

CSP was measured using a suprathreshold pulse (140% of RMT) delivered while participants maintained a voluntary isometric contraction of the abductor pollicis brevis muscle at approximately 20% of maximum contraction. The resulting silent period duration from MEP onset to return of EMG activity was averaged across 10 trials.

PAS was assessed using stimulation of the right median nerve paired with a suprathreshold TMS test pulse at an ISI of 25 ms. One hundred and eighty pairs of stimuli were delivered at 0.1 Hz. Twenty TMS pulses at 0.1 Hz were delivered before PAS and at 0-, 15-, 30-, and 60-minutes post-PAS, and resulting MEP amplitudes were measured. Ratios of average and maximum post-PAS MEP amplitudes to pre-PAS MEP amplitudes were calculated. Participants were asked to count the number of peripheral nerve stimulations on the target hand during PAS, as attention affects PAS-induced plasticity. This count was recorded randomly 8 times during the assessment of PAS and used to determine an inattention score.

### Genotyping and gene-based analyses

Genome-wide genotyping was performed using the Illumina PsychArray Beadchip v1.3 endorsed by the Psychiatric Genomics Consortium for participants from both trials. We applied to samples from both trials the quality control (QC) procedure and imputation pipeline reported previously in the first trial [42]. In the second trial, QC was also performed following the standard protocol by Anderson et al. [43] and two participants were removed due to excessive heterozygosity and genotyping missingness. 3827 variants were removed due to missingness, and 266,057 variants with <1% minor allele frequency (MAF) were excluded. 301,495 variants and 34 participants in the second trial entered genome-wide imputation. According to the *genipe* pipeline, prephasing was first conducted using SHAPEIT2 and 1000 Genomes Phase 3 reference panel, followed by imputation using IMPUTE v2.2 in 5-Mb segments per chromosome [44–47]. The final data were imputed with an information threshold of ≥0.7, a completion rate of ≥0.95, and an imputation probability threshold ≥0.9, resulting in 5,067,675 SNPs after imputation. Out of the final imputed variants, 3,882,161 variants (76.6%) with a MAF ≥ 0.05 were used in the analyses described below. After checking the availability of genetic and TMS data, 45 participants from the first trial and 4,309,635 variants were merged with imputed samples from the second trial. The final merged data file contained 79 participants and 3,331,137 variants.

Genome-wide association analysis was conducted on the merged data using PLINK v2.0 [48]. We fitted a linear regression model for each of the phenotypes (SICI at 2 ms, ICF at 10 ms, maximum PAS ratio, average PAS ratio, RMT, and CSP), adjusted for age, MADRS baseline score, and the first three principal components of ancestry inference. Sensitivity analyses were conducted that included sex as an additional covariate. GWAS summary statistics were then utilized to conduct *MAGMA* gene-based analyses using *FUMA* with default parameters [49, 50]. Using the 1000 Genome Project Phase 3 (2504 individuals, approximately 84 million SNPs) as the reference panel, 3,331,137 SNPs were assigned to 18,580 genes within a 10 kb window [46]. The genome-wide significance level after correcting for the number of genes was 0.05/18,580 = 2.691 × 10^−6^. In MAGMA analyses implanted in FUMA, the gene-based Z-score is calculated based on a SNP-wide mean model and we used the Z-score to infer the direction of gene association. To check our hypothesis about the association between the *BDNF* gene and cortical physiological changes in LLD, we looked for BDNF among the *MAGMA* results. After *MAGMA* gene-based analyses, we conducted gene-set enrichment analysis for SICI and CSP, which had significantly associated genes. We used *FUMA* with default settings and background genes mapped by *MAGMA* to determine whether there was any overlap between the top 20 associated genes and pre-defined gene sets relevant to a specific phenotype.

## Results

See Table 1 for the sociodemographic and clinical characteristics of the 79 participants included in the GWAS analyses and the subsequent gene-based analyses.

**Table 1:** Sociodemographic, clinical, and neurophysiological characteristics of the participants.

Characteristic	Median [IQR] or frequency (%)
Age	65 [62, 69.9]
Sex (Male, %)	29 (36.7%)
Ethnicity (European, %)	76 (96.2%)
MADRS score	26 [22, 30]
SICI 2 ms	0.51 [0.35, 0.76]
ICF 10 ms	1.67 [1.30, 2.37]
Average PAS ratio	1.14 [0.77, 1.44]
Maximum PAS ratio	1.55 [1.02, 2.06]
CSP	0.12 [0.10, 0.15]
RMT	47 [41, 53.5]

*CSP* cortical silent period, *ICF* intracortical facilitation, *IQR* interquartile range, *MADRS* Montgomery-Åsberg Depression Rating Scale, *PAS* paired associative stimulation, *RMT* resting motor threshold, *SICI* short interval intracortical inhibition.

### RMT

In the 79 participants with RMT data, among the mapped 18,580 genes, none of the 1154 nominally associated genes at the threshold of *p* < 0.05 reached genome-wide significance (Supplementary Fig. 1). The top association was the *MRPS31* gene (Mitochondrial Ribosomal Protein S31, Z = 4.19, *p* = 1.41 × 10^−5^; Supplementary Table 1).

### SICI

**Fig. 1:**
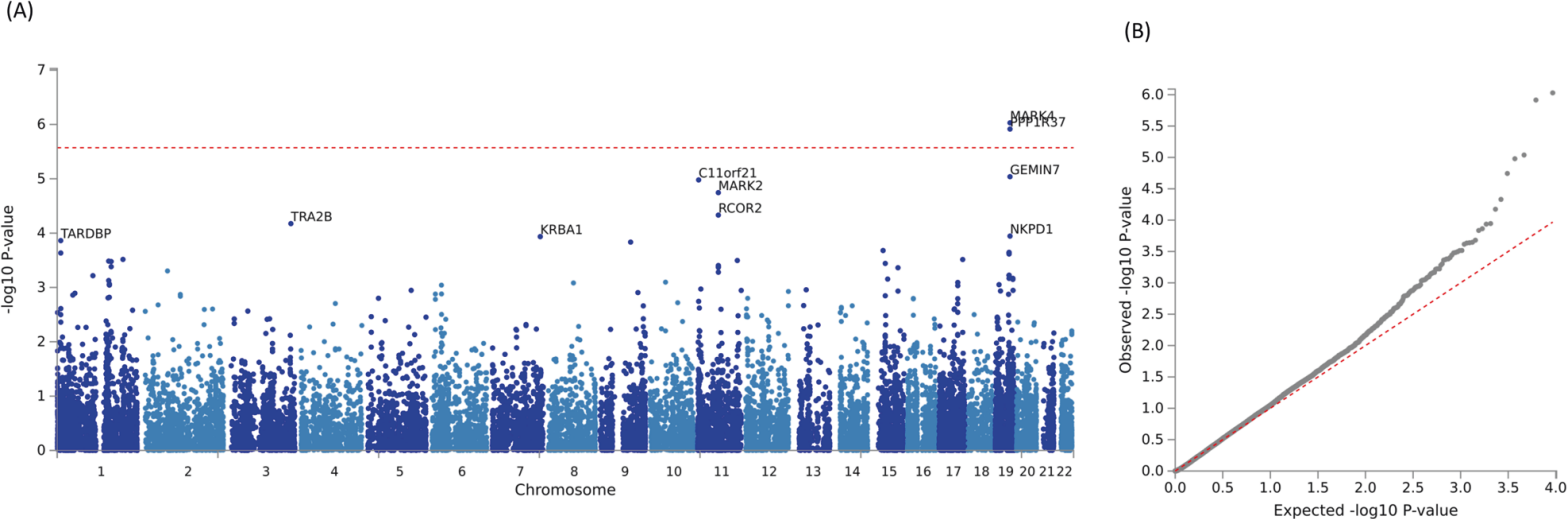
Gene-based analyses results for SICI. **A** Gene Manhattan plot. **B** Gene QQ (quantile-quantile) plot. Note: The dotted red line in (**A**) is the genome-wide significant threshold *p* = 2.691 × 10^−6^. MARK4 Microtubule Affinity Regulating Kinase 4, PPP1R37 Protein Phosphatase 1 Regulatory Subunit 37, GEMIN7 Gem Nuclear Organelle Associated Protein 7, C11orf21 Chromosome 11 Open Reading Frame 21, MARK2 Microtubule Affinity Regulating Kinase 1, RCOR2 REST Corepressor 2, TRA2B Transformer 2 Beta Homolog, NKPD1 NTPase KAP Family P-Loop Domain Containing 1, KRBA1 KRAB-A Domain Containing 1, TARDBP TAR DNA Binding Protein.

In the 78 participants with SICI data, *MARK4* (Microtubule Affinity Regulating Kinase 4, Z = 4.77, *p* = 9.35 × 10^−7^) and *PPP1R37* (Protein Phosphatase 1 Regulatory Subunit 37, Z = 4.71, *p* = 1.22 × 10^−6^) were positively associated with the SICI ratio with genome-wide significance (Fig. 1, Table 2). Among the independent SNPs assigned to *MARK4* and *PPP1R37*, the top associated SNP was rs75918199 in chromosome 19 (β = 0.50 [0.30, 0.71], *p* = 8.66 × 10^−6^). 1122 genes were nominally associated with the phenotype (*p* < 0.05). Among the top 20 associated genes, the gene-set enrichment analysis showed that the GWAS catalog gene-set of Alzheimer’s disease or pleiotropy had 4 genes (*GEMIN7, MARK4*, *PPP1R37*, and *NKPD1*) overlapped (*p* = 1.74 × 10^−7^, adjusted-*p* = 3.16 × 10^−4^; Supplementary Table 5).

**Table 2:** Summary statistics of top 10 genes associated with SICI ratio.

Gene	Chromosome	Start position	Stop position	N_SNPs_	Z-Statistics	*p*-value
MARK4	19	45572546	45818541	225	4.77	9.35 × 10^−7^
PPP1R37	19	45584654	45661335	157	4.71	1.22 × 10^−6^
GEMIN7	19	45572453	45604782	91	4.28	9.16 × 10^−6^
C11orf21	11	2306875	2334279	50	4.25	1.05 × 10^−5^
MARK2	11	63596400	63688491	62	4.13	1.80 × 10^−5^
RCOR2	11	63668693	63694316	11	3.91	4.67 × 10^−5^
TRA2B	3	185623694	185665924	49	3.82	6.70 × 10^−5^
NKPD1	19	45643008	45673408	9	3.69	1.13 × 10^−4^
KRBA1	7	149401872	149441664	35	3.68	1.16 × 10^−4^
TARDBP	1	11062414	11095796	35	3.64	1.37 × 10^−4^

*MARK4* Microtubule Affinity Regulating Kinase 4, *PPP1R37* Protein Phosphatase 1 Regulatory Subunit 37, *GEMIN7* Gem Nuclear Organelle Associated Protein 7, *C11orf21* Chromosome 11 Open Reading Frame 21, *MARK2* Microtubule Affinity Regulating Kinase 1, *RCOR2* REST Corepressor 2, *TRA2B* Transformer 2 Beta Homolog, *NKPD1* NTPase KAP Family P-Loop Domain Containing 1, *KRBA1* KRAB-A Domain Containing 1, *TARDBP* TAR DNA Binding Protein.

### CSP

**Fig. 2:**
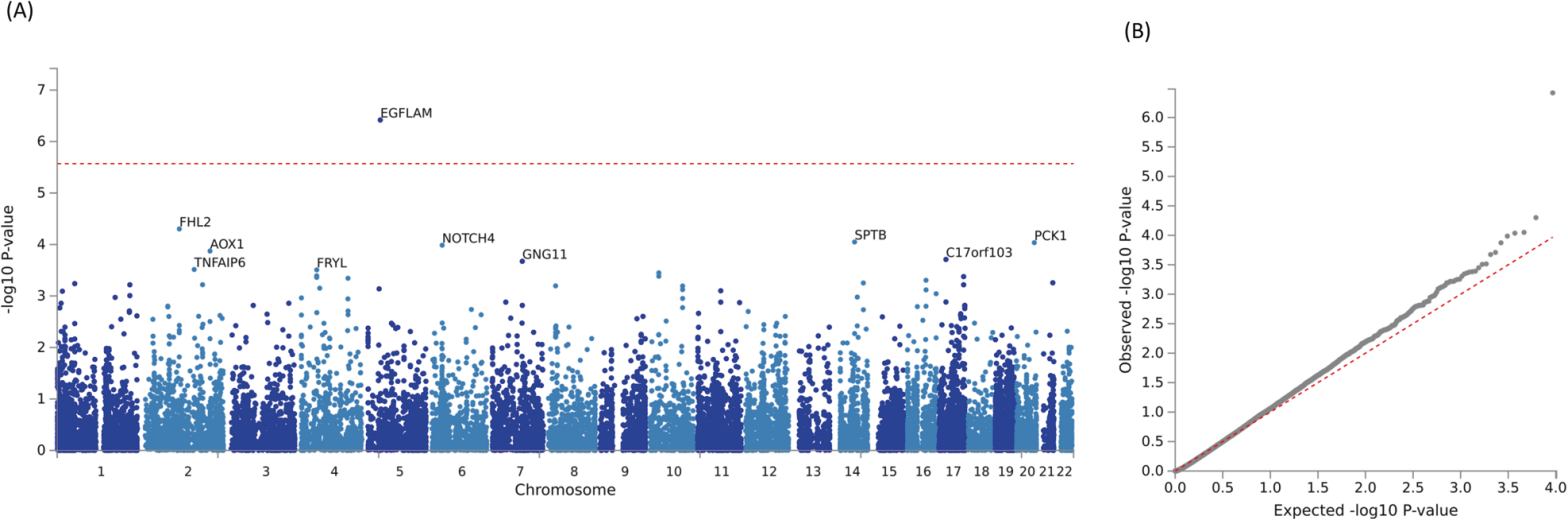
Gene-based analyses results for CSP. **A** Gene Manhattan plot. **B** Gene QQ (quantile-quantile) plot. *Note*: The dotted red line in (**A**) is the genome-wide significant threshold *p* = 2.691 × 10^−6^. EGFLAM EGF Like - Fibronectin Type III and Laminin G Domains, FHL2 Four And A Half LIM Domains 2, SPTB Spectrin Beta Erythrocytic, PCK1 Phosphoenolpyruvate Carboxykinase 1, NOTCH4 Notch Receptor 4, AOX1 Aldehyde Oxidase 1, C17orf103 N-Acetyltransferase Domain Containing 1, GNG11 G Protein Subunit Gamma 11, TNFAIP6 TNF Alpha Induced Protein 6, FRYL FRY Like Transcription Coactivator.

In 76 participants with CSP data, *EGFLAM* (EGF-like Fibronectin Type III and Laminin G Domains, Z = 4.94, *p* = 3.82 × 10^−7^) was positively associated with CSP with genome-wide significance (Fig. 2, Table 3). Among the independent SNPs assigned to *EGFLAM*, the top associated SNP was rs2561139 in chromosome 5 (β = −0.02 [−0.03, −0.01], *p* = 7.85 × 10^−6^). 1126 genes were nominally associated with the phenotype (*p* < 0.05). None of the top 20 associated genes were overrepresented in the pre-defined GWAS catalog reported gene sets.

**Table 3:** Summary statistics of top 10 genes associated with CSP.

Gene	Chromosome	Start position	Stop position	N_SNPs_	Z-Statistics	*p*-value
EGFLAM	5	38248511	38475123	205	4.94	3.82 × 10^−7^
FHL2	2	105964169	106064970	106	3.89	5.01 × 10^−5^
SPTB	14	65203002	65356601	225	3.75	8.91 × 10^−5^
PCK1	20	56126136	56151513	29	3.74	9.20 × 10^−5^
NOTCH4	6	32152620	32201844	166	3.71	1.03 × 10^−4^
AOX1	2	201440591	201551787	150	3.64	1.34 × 10^−4^
C17orf103	17	21132183	21166722	16	3.55	1.95 × 10^−4^
GNG11	7	93541011	93567922	27	3.53	2.12 × 10^−4^
TNFAIP6	2	152204106	152246560	51	3.43	3.06 × 10^−4^
FRYL	4	48489378	48792339	164	3.42	3.11 × 10^−4^

*EGFLAM* EGF-Like Fibronectin Type III and Laminin G Domains, *FHL2* Four And A Half LIM Domains 2, *SPTB* Spectrin Beta Erythrocytic, *PCK1* Phosphoenolpyruvate Carboxykinase 1, *NOTCH4* Notch Receptor 4, *AOX1* Aldehyde Oxidase 1, *C17orf103* N-Acetyltransferase Domain Containing 1, *GNG11* G Protein Subunit Gamma 11, *TNFAIP6* TNF Alpha Induced Protein 6, *FRYL* FRY Like Transcription Coactivator.

### ICF

In the 77 participants with ICF data, none of the 11,161 nominally associated genes reached genome-wide significance (Supplementary Fig. 2). The top association was *SMIM17* gene (Small Integral Membrane Protein 17, Z = 4.30, *p* = 8.65 × 10^−6^; Supplementary Table 2).

### Average PAS ratio

In the 63 participants with a PAS ratio, none of the 1067 nominally associated genes reached genome-wide significance (Supplementary Fig. 3). The top association was the *TRIM58* gene (Tripartite Motif Containing 58, Z = 3.72, *p* = 9.95 × 10^−5^; Supplementary Table 3).

### Maximum PAS ratio

In the 63 participants with a PAS ratio, none of the 1107 nominally associated genes reached genome-wide significance (Supplementary Fig. 4). The top association was the *PRKAG3* gene (Protein Kinase AMP-Activated Non-Catalytic Subunit Gamma 3, Z = 3.99, *p* = 3.37 × 10^−5^; Supplementary Table 4).

### Sensitivity Analyses

With sex as an additional covariate, *PPP1R37* was not significantly associated with SICI (Z = 4.53, *p* = 2.90 × 10^−6^). *MARK4* remained associated with SICI (Z = 4.58, *p* = 2.38 × 10^−6^) and *EGFLAM* remained associated with CSP (Z = 4.94, *p* = 3.99 × 10^−7^) with genome-wide significance. There were no other significant associations.

## Discussion

We used exploratory GWAS and gene-based analyses to assess the association between genes and TMS measures of cortical inhibition, facilitation, or LTP-like plasticity in LLD. We had three key findings. First, *MARK4* (which encodes microtubule affinity-regulating kinase 4) and *PPP1R37* (which encodes protein phosphatase 1 regulatory subunit 37) showed genome-wide significant associations with SICI, an indirect measure of GABA_A_ receptor-mediated cortical inhibition. Second, *EGFLAM* (which encodes EGF-like fibronectin type III and laminin G domains) showed genome-wide significant association with CSP, an indirect measure of GABA_B_ receptor-mediated cortical inhibition. Third, no genes showed a genome-wide significant association with RMT, ICF, or PAS. These findings suggest there may be genetic influences on cortical inhibition in older adults with LLD. Further research is indicated to determine whether cortical inhibition may serve as a biomarker to improve diagnostic precision and treatment selection in this patient population.

SICI is believed to be an indirect measure of GABA_A_ inhibitory activity [10]. *MARK4* and *PPP1R37* were both associated with decreased SICI in our sample. *MARK4* encodes a protein that may be involved in regulation of microtubule networks in neurons [51]. Increased expression of *MARK4* has been shown in Alzheimer’s disease (AD), and in vitro studies suggest *MARK4* can potentiate tau aggregation [51]. Based on gene enrichment testing in FUMA, both *MARK4* and *PPP1R37* have been reported in “Alzheimer’s disease or HDL levels” in the GWAS catalog (see Supplementary Fig. 6). A de novo mutation of *MARK4* has been associated with the production of abnormally phosphorylated tau [51]. In vivo studies in mice suggest tau expression may be associated with synaptic plasticity deficits [52]. Elevated tau has also been associated with depression, and recent literature suggests that LLD has a different distribution of tau compared to AD [53, 54]. In theory, tau aggregation could inhibit activity of GABAergic neurons, like its effects on hippocampal neurons in AD [55]. Furthermore, *MARK4* has also been shown to be upregulated in ischemic axonal injury [56]. Cerebrovascular disease (CVD) may also contribute to LLD, and CVD has also been linked to AD [57, 58].

*PPP1R37* encodes for a protein believed to inhibit phosphatase activity of protein phosphatase 1 (PP1) complexes [59]. Limited literature exists on the function of this protein. A transcriptome-wide analysis found an association of differential expression of *PPP1R37* in hippocampal tissue with AD compared to controls [60]. Post-mortem studies of patients with AD suggest that PP1 is involved in regulation of tau dephosphorylation [61]. Experiments in mice suggest PP1 activity mediates effects of β-amyloid on synaptic plasticity in AD and PP1 has a role in long-term depression of neuronal activity [61]. Thus, variance in expression of *PPP1R37* could lead to aberrant phosphorylation of neurotoxic aggregates, with subsequent effects on GABAergic function. Of note, *PPP1R37* was not significantly associated with SICI in sensitivity analyses including sex as an additional covariate. Sex was not included as a covariate in the primary analyses due to limited sample size, however, there is one existing study that suggests biological males may have reduced SICI compared to females [62]. More studies are needed to characterize the role of sex in relation to depression and cortical physiology changes.

Existing literature reports a significant association between LLD and AD, with LLD being a risk factor or prodrome for AD [63]. Several biological mechanisms may overlap between depression and AD, including: inflammatory changes, CVD, decreased neurotrophic factors, and deposits of β-amyloid plaques [63]. The potential associations of both *MARK4* and *PPP1R37* with AD-related processes is supportive of a link between LLD and AD. The age-by-disease interaction hypothesis of LLD suggests age-related changes in gene expression in the brain may increase susceptibility to multiple neurodegenerative disorders simultaneously [64]. A previous study showed that deficits in cortical inhibition measured with SICI can occur with aging, independent of depression status [3]. Given that the present study is a cross-sectional analysis of cortical physiology in LLD and there is no age-matched control group, we cannot assess whether the potential effects of *MARK4* and *PPP1R37* are related to depression, aging, or both.

CSP is believed to be an indirect measure of GABA_B_ inhibitory activity [11]. *EGFLAM* was associated with increased CSP in our sample. *EGFLAM* encodes a protein believed to enable calcium ion and glycosaminoglycan binding activity [65]. Previous GWAS studies suggest *EGFLAM* is associated with mathematical ability, educational attainment, and SSRI response [66–68]. There are no prior studies that have investigated the effect of *EGFLAM* on TMS cortical physiology measures. More studies are needed to clarify if *EGFLAM* is associated with cortical physiology.

Identifying predictors of response is important to optimize treatment selection for LLD. In a previous study, a combination of TMS measures of cortical excitability, with clinical and demographic data predicted response to venlafaxine treatment in LLD with 73% accuracy [69]. The combination of TMS measures with additional biomarkers, such as genetic data, may be useful for the development of models with higher accuracy. As cortical physiological processes are influenced by both neurostimulation and pharmacotherapy, further investigation to characterize the genetic determinants of cortical physiology in LLD may help identify genetic biomarkers of treatment response.

In our study, *BDNF* did not show a genome-wide significant association with SICI, CSP, ICF, RMT, or PAS. The association of *BDNF* genotype with TMS cortical physiology measures has been previously investigated. In a few studies in healthy participants, there were no differences in SICI, ICF, CSP, or RMT based on *BDNF* genotype [19–22]. In three small studies in young healthy participants (*N* = 18–32), there was an increase in post-PAS MEP amplitudes in those with the *BDNF* valine/valine genotype, but not in those with the Val66Met SNP [23–25]. One case-control study found a significant interaction between BDNF genotype and catechol-O-methyltransferase genotype in PAS response, suggesting there are likely polygenic determinants of LTP-like plasticity [70]. A case-control study of 23 participants with MDD matched to 23 healthy controls reported greater post-PAS MEP amplitudes in healthy controls than participants with depression, though there was no statistically significant effect of *BDNF* genotype on response to PAS [71]. There are also contradictory reports on the effect of the Val66Met SNP on TMS cortical physiology measures in response to interventions such as motor training, intermittent theta-burst stimulation, and transcranial direct current stimulation [19–22]. In light of our findings, based on lack of agreement in studies, more studies are needed to clarify whether *BDNF* is associated with cortical physiology in younger and older adults with and without depression.

A limited number of prior studies have assessed the association of other genes with TMS cortical physiology measures. A randomized crossover trial of 92 healthy participants found no effect of the *SCN1A* rs3812717 polymorphism on SICI, ICF, CSP, or RMT, however, the *SCNIA* genotype modulated the effect of carbamazepine on CSP [26]. A cross-sectional analysis of 77 healthy participants found two common *TRPV1* SNPs do not have an effect on SICI, ICF, CSP, or RMT [27]. A small randomized crossover trial in children with Attention-deficit/hyperactivity disorder (ADHD) found that genetic variation in the dopamine transporter (*DAT1*) was not associated with baseline differences in SICI or ICF, but *DAT1* genotype influenced the effect of stimulant administration on these measures [72]. A small exploratory study of eight healthy participants suggested allelic variation in the serotonin transporter promoter (*5-HTTLPR*) is associated with differential cortical excitability at baseline or in response to citalopram administration [28]. In addition, a case-control study in 24 participants with mild cognitive impairment compared to 24 age-matched controls found that *APOE* genotype was not correlated with response to PAS [29]. There are no prior studies that have investigated the effect of genes on TMS cortical physiology measures in LLD specifically. In our study, *SCN1A*, *TRPV1*, *DAT1*, and *APOE* did not show an association with TMS cortical physiology measures, and more studies are needed to clarify whether these genes are associated with cortical physiology processes.

Some strengths of our analysis include comprehensive clinical characterization of the patient sample and integration of several biological measures related to genome-wide genotyping and TMS neurophysiology. LLD is a heterogenous disorder in relation to pathophysiology, clinical phenotype, and intervention response. Recent literature suggests the importance of transdisciplinary approaches to research in psychiatry, as biological systems rarely act in isolation [73]. Comprehensive clinical and biological characterization of patients receiving treatment for LLD is needed given the complexity of depression and aging.

There were also several limitations in our analysis. First and foremost, our sample size was small, increasing the risk of type II error and potentially preventing us from finding true associations. Second, our results were specific to the motor cortex, and it is unclear how they would relate to other cortical regions. Future studies should assess genetic correlations of TMS measures in the dorsolateral prefrontal cortex directly, using TMS-EEG [74]. We were unable to assess for epistasis, that has been shown to have salient impacts on depression phenotypes [75, 76]. We were also unable to assess for causal associations of genes with the TMS processes, as this would require functional characterization of genotypes. As there is no control group in the analysis, we are unable to distinguish whether the identified SNPs are more relevant to depression or aging.

Furthermore, additional covariates that may impact cortical physiology, such as prescribed medications and comorbidities, were not accounted for in this analysis. Limited evidence suggests that single doses of specific serotonin reuptake inhibitors and norepinephrine reuptake inhibitors may have transient effects on cortical physiology [31–33]. Several studies suggest that administration of dopamine agonists and antagonists can modulate cortical inhibition, facilitation, and plasticity [34, 35, 77]. The impacts of other psychotropic classes, treatment duration, and medication interactions on cortical physiology have not been well-characterized. In addition, medical comorbidities are relatively common in older adults. Prior studies show that following stroke there may be reduced SICI, which may persist throughout the recovery course [78, 79]. The impact of other comorbidities, such as cerebrovascular disease or other neurological conditions, on cortical physiology is not well-defined. More studies are needed to elucidate the role of medications and comorbidities in cortical physiology.

In conclusion, our results suggest there may be genetic influences on cortical physiology in LLD. *MARK4* and *PPP1R37* (two genes that have been linked to AD), and *EGFLAM*, may influence cortical inhibition in LLD. Replication with larger sample sizes and functional analyses of relevant SNPs is needed to determine possible causal associations of genes with TMS measures. With an increasing number of treatment options available for LLD, it is important to characterize the influences of genetics on cortical neurophysiology. This could facilitate optimization of treatment selection and advancement of personalized medicine for LLD.

## Supplementary information

Supplementary Information

Supplementary Figure 1

Supplementary Figure 2

Supplementary Figure 3

Supplementary Figure 4

Supplementary Figure 5

Supplementary Figure 6
