# Supplementary material for: Exploratory genome-wide analyses of cortical inhibition, facilitation, and plasticity in late-life depression
Source: Transl Psychiatry. 2023 Jun 30;13:234. doi: 10.1038/s41398-023-02532-0 (PMC10313655; doi:10.1038/s41398-023-02532-0)
Supplement: Supplementary file 1 — Supplementary Information [file 41398_2023_2532_MOESM1_ESM.docx]

**SUPPLEMENTARY INFORMATION**

**SUPPLEMENTARY FIGURE LEGENDS**

**Supplementary Figure 1. Gene-based analyses results for RMT**

1. Gene Manhattan plot
2. Gene QQ (quantile-quantile) plot

**Note:** The dotted red line in Figure a is the genome-wide significant threshold p = 2.691 × 10^-6^.

**Abbreviations:** MRPS31, Mitochondrial Ribosomal Protein S31; SYNC, Syncoilin; C8orf47, Chromosome 8 Open Reading Frame 74; SLC4A2, Solute Carrier Family 4 Member 2; CDK5, Cyclin Dependent Kinase 5; ELOVL1, ELOVL Fatty Acid Elongase 1; CDKL1, Cyclin Dependent Kinase Like 1; PPM1H, Protein Phosphatase Mg^2+^/Mn^2+^ Dependent 1H; LSS, Lanosterol Synthase.

**Supplementary Figure 2. Gene-based analyses results for ICF**

1. Gene Manhattan plot
2. Gene QQ (quantile-quantile) plot

Note: The dotted red line in Figure a is the genome-wide significant threshold p = 2.691 × 10^-6^.

**Abbreviations:** SMIM17, Small Integral Membrane Protein 17; NHSL1, NHL Like 1; PDGFC, Platelet Derived Growth Factor C; ZCRB1, Zinc Finger CCHC-Type And RNA Binding Motif Containing 1; PPHLN1, Periphilin 1; MBIP, MAP3K12 Binding Inhibitory Protein 1; MTHFS, Methenyltetrahydrofolate Synthetase; LNX2, Ligand Of Numb-Protein X 2; CCNA1, Cyclin A1.

**Supplementary Figure 3. Gene-based analyses results for average PAS ratio**

1. Gene Manhattan plot
2. Gene QQ (quantile-quantile) plot

Note: The dotted red line in Figure a is the genome-wide significant threshold p = 2.691 × 10^-6^.

**Abbreviations:** TRIM58, Tripartite Motif Containing 58; PRKAG3, Protein Kinase AMP-Activated Non-Catalytic Subunit Gamma 3; CEACAM16, CEA Cell Adhesion Molecule 16 Tectorial Membrane Component; IFFO1, Intermediate Filament Family Orphan 1; OR2W3, Olfactory Receptor Family 2 Subfamily W Member 3; KCNV1, Potassium Voltage-Gated Channel Modifier Subfamily V Member 1; DPEP3, Dipeptidase 3; CTCF, CCCTC-Binding Factor; TBC1D32, TBC1 Domain Family Member 32; GAPDH, Glyceraldehyde-3-Phosphate Dehydrogenase.

**Supplementary Figure 4. Gene-based analyses results for maximum PAS ratio**

1. Gene Manhattan plot
2. Gene QQ (quantile-quantile) plot

Note: The dotted red line in Figure a is the genome-wide significant threshold p = 2.691 × 10^-6^.

**Abbreviations:** PRKAG3, Protein Kinase AMP-Activated Non-Catalytic Subunit Gamma 3; OAF, Out At First Homolog; DPEP3, Dipeptidase 3; CEACAM16, CEA Cell Adhesion Molecule 16 Tectorial Membrane Component; CETN3, Centrin 3; ARHGAP26, Rho GTPase Activating Protein 26; OR2W3, Olfactory Receptor Family 2 Subfamily W Member 3; IFI27, Interferon Alpha Inducible Protein 27; TRIM58, Tripartite Motif Containing 58; CTCF, CCCTC-Binding Factor.

**Supplementary Figure 5. Expression Heatmap of Top 20 Associated Genes**

a. Gene expression heatmap for the top 20 associated genes of SICI

b. Gene expression heatmap for the top 20 associated genes of CSP

Note: Gene expression heatmap was generated for the top 20 associated genes for SICI and CSP, using default parameters in FUMA. The data set we used was GTEx v8 54 tissue types. Higher expression value/red expression color indicates higher expression in a specific tissue.

**Supplementary Figure 6. Enrichment Results in Gene Sets for the Top 20 Associated Genes of SICI**

Note: Gene enrichment test was performed in FUMA to see if any top 20 associated genes were reported in previous genomic analyses. Only Gene sets with Benjamini-Hochberg (FDR)-adjusted p-value < 0.05 were considered significant and displayed.

**SUPPLEMENTARY TABLES**

**Supplementary Table 1. Summary Statistics of Top 10 Genes Associated with RMT**

| **Gene** | **Chromosome** | **Start Position** | **Stop Position** | **N_SNPs_** | **Z-Statistics** | **p-value** |
| --- | --- | --- | --- | --- | --- | --- |
| MRPS31 | 13 | 41293432 | 41355309 | 54 | 4.19 | 1.41 × 10^-5^ |
| SYNC | 1 | 33135507 | 33179197 | 14 | 4.12 | 1.88 × 10^-5^ |
| C8orf74 | 8 | 10520147 | 10568103 | 22 | 3.92 | 4.43 × 10^-5^ |
| SLC4A2 | 4 | 150744297 | 150783614 | 22 | 3.74 | 9.09 × 10^-5^ |
| CDK5 | 7 | 150740899 | 150765617 | 29 | 3.62 | 1.48 × 10^-4^ |
| ELOVL1 | 16 | 43819068 | 43843696 | 21 | 3.59 | 1.67 × 10^-4^ |
| CDKL1 | 7 | 50786310 | 50893179 | 112 | 3.57 | 1.80 × 10^-4^ |
| PPM1H | 7 | 63027762 | 63338817 | 222 | 3.57 | 1.81 × 10^-4^ |
| CTD-2144E22.5 | 20 | 34246507 | 34267278 | 1 | 3.55 | 1.95 × 10^-4^ |
| LSS | 1 | 47598055 | 47658738 | 149 | 3.54 | 1.97 × 10^-4^ |

**Abbreviations:** MRPS31, Mitochondrial Ribosomal Protein S31; SYNC, Syncoilin; C8orf47, Chromosome 8 Open Reading Frame 74; SLC4A2, Solute Carrier Family 4 Member 2; CDK5, Cyclin Dependent Kinase 5; ELOVL1, ELOVL Fatty Acid Elongase 1; CDKL1, Cyclin Dependent Kinase Like 1; PPM1H, Protein Phosphatase Mg^2+^/Mn^2+^ Dependent 1H; LSS, Lanosterol Synthase.

**Supplementary Table 2.** **Summary Statistics of Top 10 Genes Associated with ICF**

| **Gene** | **Chromosome** | **Start Position** | **Stop Position** | **N_SNPs_** | **Z-Statistics** | **p-value** |
| --- | --- | --- | --- | --- | --- | --- |
| SMIM17 | 19 | 57144513 | 57177134 | 23 | 4.30 | 8.65 × 10^-6^ |
| NHSL1 | 6 | 138733180 | 139023708 | 192 | 4.25 | 1.08 × 10^-5^ |
| PDGFC | 4 | 157671606 | 157902546 | 137 | 4.20 | 1.35 × 10^-5^ |
| ZCRB1 | 12 | 42695880 | 42729920 | 51 | 4.13 | 1.78 × 10^-5^ |
| PPHLN1 | 12 | 42622249 | 42863517 | 425 | 4.06 | 2.48 × 10^-5^ |
| MBIP | 14 | 36757770 | 36799882 | 1 | 3.94 | 4.05 × 10^-5^ |
| MTHFS | 15 | 80115927 | 80199721 | 193 | 3.93 | 4.21 × 10^-5^ |
| LNX2 | 13 | 28110050 | 28204541 | 188 | 3.91 | 4.53 × 10^-5^ |
| CCNA1 | 13 | 36995967 | 37027019 | 27 | 3.67 | 1.21 × 10^-4^ |
| AC020629.1 | 12 | 42614050 | 42638075 | 22 | 3.64 | 1.35 × 10^-5^ |

**Abbreviations:** SMIM17, Small Integral Membrane Protein 17; NHSL1, NHL Like 1; PDGFC, Platelet Derived Growth Factor C; ZCRB1, Zinc Finger CCHC-Type And RNA Binding Motif Containing 1; PPHLN1, Periphilin 1; MBIP, MAP3K12 Binding Inhibitory Protein 1; MTHFS, Methenyltetrahydrofolate Synthetase; LNX2, Ligand Of Numb-Protein X 2; CCNA1, Cyclin A1.

**Supplementary Table 3.** **Summary Statistics of Top 10 Genes Associated with Average PAS Ratio**

| **Gene** | **Chromosome** | **Start Position** | **Stop Position** | **N_SNPs_** | **Z-Statistics** | **p-value** |
| --- | --- | --- | --- | --- | --- | --- |
| TRIM58 | 1 | 248010501 | 248051507 | 77 | 3.72 | 9.95 × 10^-5^ |
| PRKAG3 | 2 | 219677106 | 219706809 | 1 | 3.65 | 1.32 × 10^-4^ |
| CEACAM16 | 19 | 45192421 | 45223986 | 1 | 3.58 | 1.74 × 10^-4^ |
| IFFO1 | 12 | 6637541 | 6675239 | 36 | 3.56 | 1.87 × 10^-4^ |
| OR2W3 | 1 | 248021277 | 248070449 | 99 | 3.54 | 1.97 × 10^-4^ |
| KCNV1 | 8 | 110965874 | 110998076 | 18 | 3.48 | 2.50 × 10^-4^ |
| DPEP3 | 16 | 67999566 | 68024732 | 6 | 3.48 | 2.55 × 10^-4^ |
| CTCF | 16 | 67586310 | 67683086 | 31 | 3.40 | 3.33 × 10^-4^ |
| TBC1D32 | 6 | 121390640 | 121665891 | 283 | 3.40 | 3.35 × 10^-4^ |
| GAPDH | 12 | 6633093 | 6657537 | 31 | 3.39 | 3.54 × 10^-4^ |

**Abbreviations:** TRIM58, Tripartite Motif Containing 58; PRKAG3, Protein Kinase AMP-Activated Non-Catalytic Subunit Gamma 3; CEACAM16, CEA Cell Adhesion Molecule 16 Tectorial Membrane Component; IFFO1, Intermediate Filament Family Orphan 1; OR2W3, Olfactory Receptor Family 2 Subfamily W Member 3; KCNV1, Potassium Voltage-Gated Channel Modifier Subfamily V Member 1; DPEP3, Dipeptidase 3; CTCF, CCCTC-Binding Factor; TBC1D32, TBC1 Domain Family Member 32; GAPDH, Glyceraldehyde-3-Phosphate Dehydrogenase.

**Supplementary Table 4. Summary Statistics of Top 10 Genes Associated with Maximum PAS Ratio**

| **Gene** | **Chromosome** | **Start Position** | **Stop Position** | **N_SNPs_** | **Z-Statistics** | **p-value** |
| --- | --- | --- | --- | --- | --- | --- |
| PRKAG3 | 2 | 219677106 | 219706809 | 1 | 3.99 | 3.37 × 10^-5^ |
| OAF | 11 | 120071475 | 120111041 | 45 | 3.62 | 1.45 × 10^-4^ |
| DPEP3 | 16 | 67999566 | 68024732 | 6 | 3.57 | 1.76 × 10^-4^ |
| CEACAM16 | 19 | 45192421 | 45223986 | 1 | 3.57 | 1.77 × 10^-4^ |
| CETN3 | 5 | 89678078 | 89715603 | 3 | 3.56 | 1.82 × 10^-4^ |
| ARHGAP26 | 5 | 142139949 | 142618576 | 261 | 3.54 | 2.02 × 10^-4^ |
| OR2W3 | 1 | 248021277 | 248070449 | 99 | 3.53 | 2.08 × 10^-4^ |
| IFI27 | 14 | 94561182 | 94593033 | 29 | 3.47 | 2.58 × 10^-4^ |
| TRIM58 | 1 | 248010501 | 248051507 | 77 | 3.46 | 2.73 × 10^-4^ |
| CTCF | 16 | 67586310 | 67683086 | 31 | 3.39 | 3.44 × 10^-4^ |

**Abbreviations:** PRKAG3, Protein Kinase AMP-Activated Non-Catalytic Subunit Gamma 3; OAF, Out At First Homolog; DPEP3, Dipeptidase 3; CEACAM16, CEA Cell Adhesion Molecule 16 Tectorial Membrane Component; CETN3, Centrin 3; ARHGAP26, Rho GTPase Activating Protein 26; OR2W3, Olfactory Receptor Family 2 Subfamily W Member 3; IFI27, Interferon Alpha Inducible Protein 27; TRIM58, Tripartite Motif Containing 58; CTCF, CCCTC-Binding Factor.

**Supplementary Table 5. Gene-set Enrichment Analyses Results for Top 20 SICI-Associated Genes**

| **Gene-set** | **N_genes_** | **N_overlapped_** | ***p*-value** | **Adjusted *p*-value** | **Overlapped Genes** |
| --- | --- | --- | --- | --- | --- |
| Alzheimer's disease or HDL levels (pleiotropy) | 52 | 4 | 1.74 × 10^-7^ | 3.16 × 10^-4^ | GEMIN7, MARK4, PPP1R37, NKPD1 |
| Body mass index x age interaction | 34 | 3 | 4.75 × 10^-6^ | 4.31 × 10^-3^ | GEMIN7, MARK4, PPP1R37 |
| Cerebrospinal fluid AB1-42 levels | 48 | 3 | 1.36 × 10^-5^ | 7.00 × 10^-3^ | GEMIN7, PPP1R37, NKPD1 |
| Alzheimer's disease or family history of Alzheimer's disease | 50 | 3 | 1.54 × 10^-5^ | 7.00 × 10^-3^ | GEMIN7, MARK4, NKPD1 |
| Body mass index (age>50) | 57 | 3 | 2.29 × 10^-5^ | 8.32 × 10^-3^ | GEMIN7, MARK4, PPP1R37 |
| Body mass index x sex x age interaction (4df test) | 86 | 3 | 7.87 × 10^-5^ | 2.38 × 10^-2^ | GEMIN7, MARK4, PPP1R37 |

**Note:** Benjamini-Hochberg (FDR) method was used to correct *p*-value.

**Abbreviations:** GEMIN7, Gem Nuclear Organelle Associated Protein 7; HDL, high-density lipoprotein; MARK4, Microtubule Affinity Regulating Kinase 4; NKPD1, NTPase KAP Family P-Loop Domain Containing 1; PPP1R37, Protein Phosphatase 1 Regulatory Subunit 37.
