# Supplementary figures and images for: Exploratory genome-wide analyses of cortical inhibition, facilitation, and plasticity in late-life depression
Source: Transl Psychiatry. 2023 Jun 30;13:234. doi: 10.1038/s41398-023-02532-0 (PMC10313655; doi:10.1038/s41398-023-02532-0)

(A)

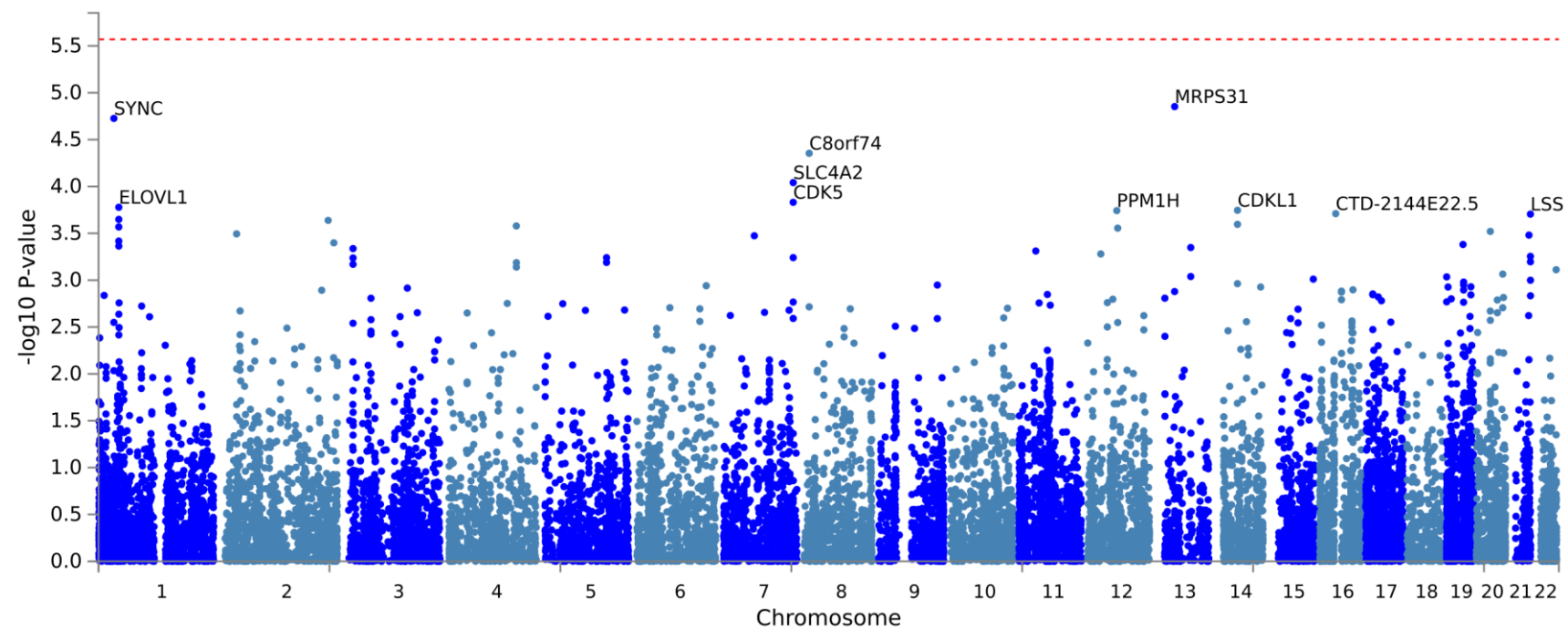

(B)

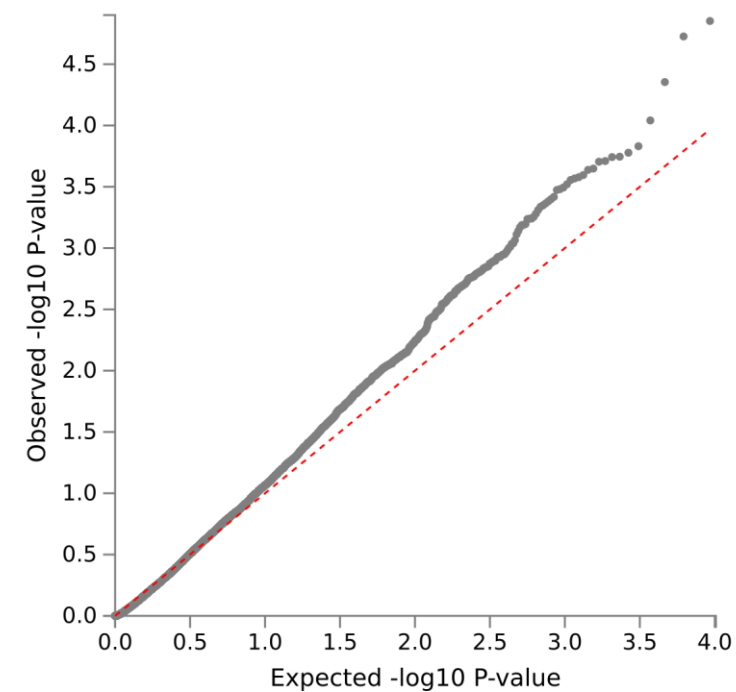

Supplement: Supplementary file 2 — Supplementary Figure 1 [file 41398_2023_2532_MOESM2_ESM.pdf]

(A)

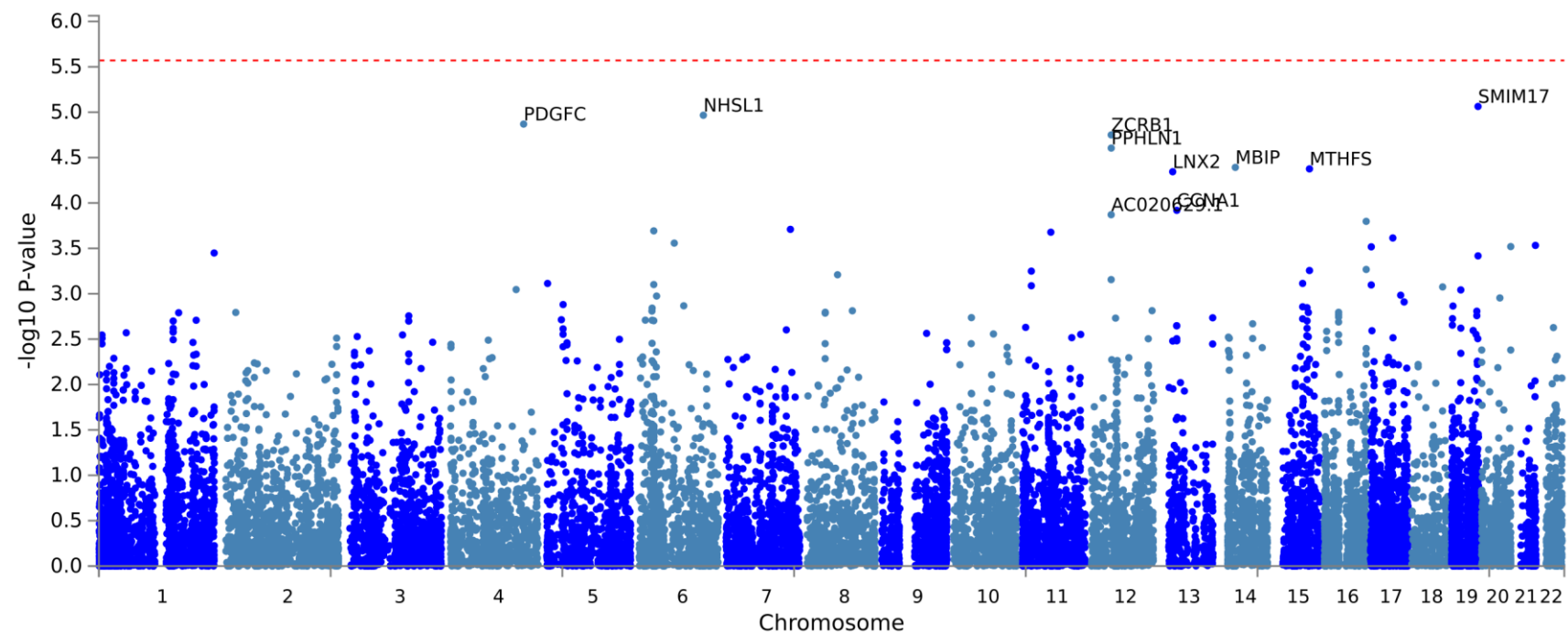

(B)

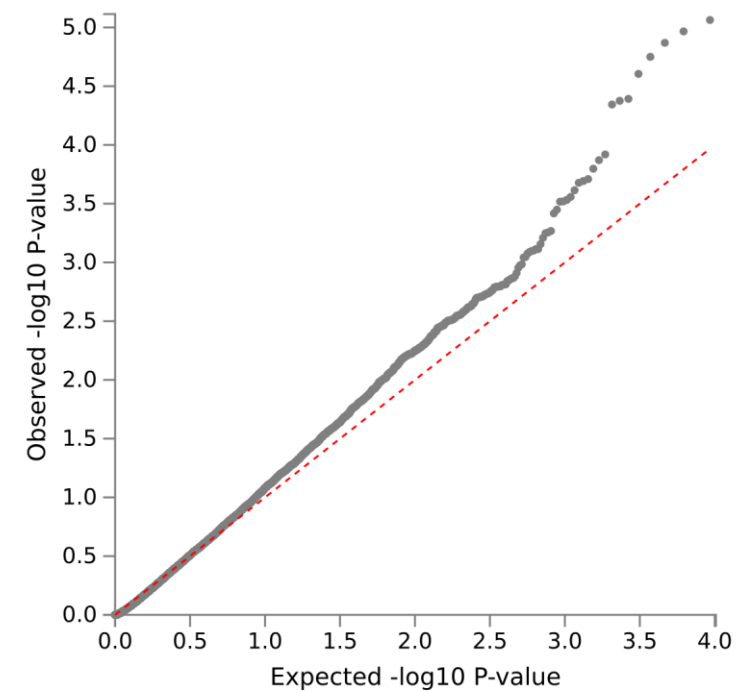

Supplement: Supplementary file 3 — Supplementary Figure 2 [file 41398_2023_2532_MOESM3_ESM.pdf]

(A)

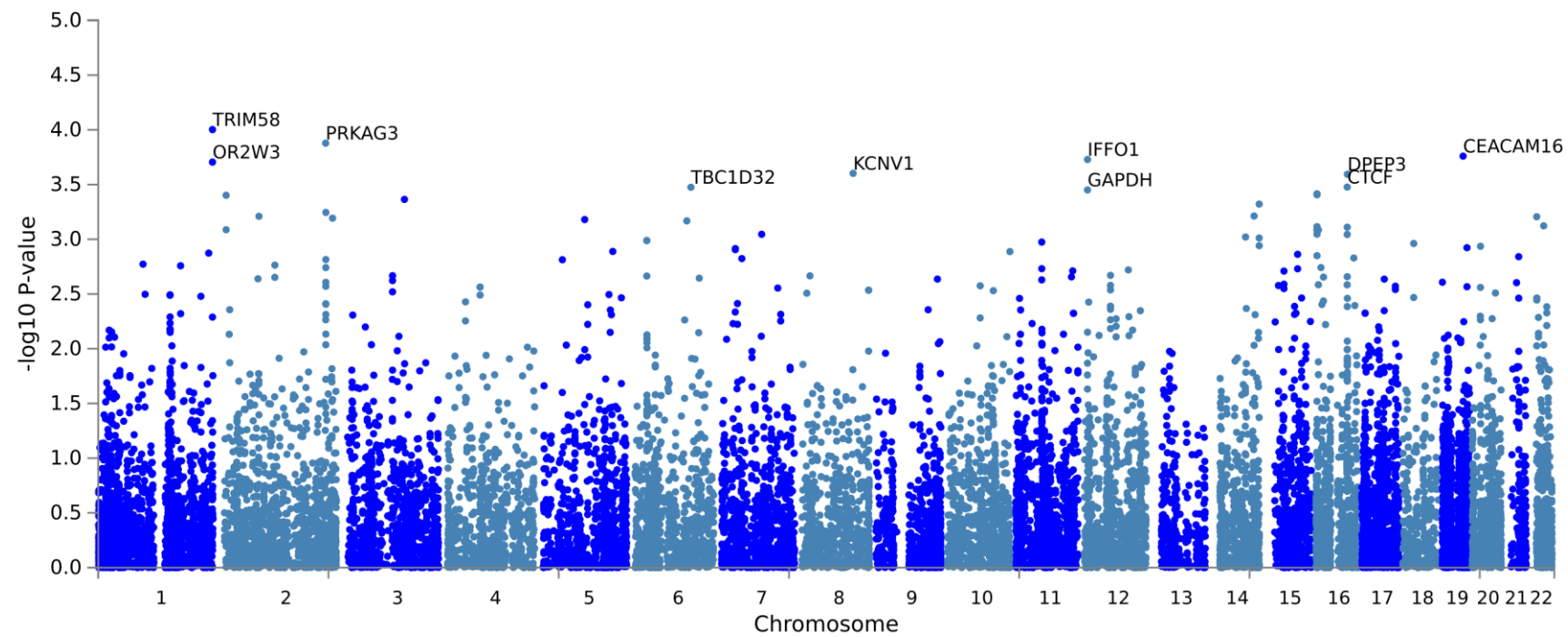

(B)

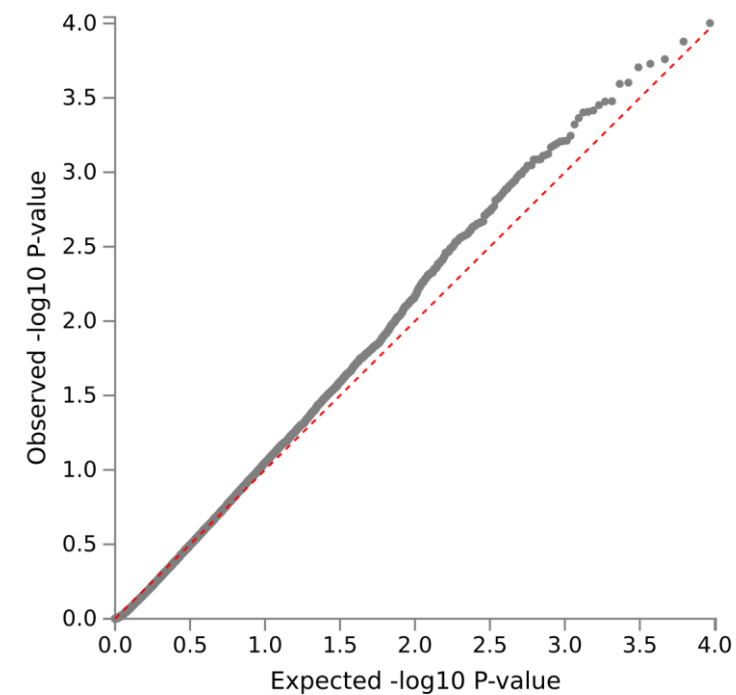

Supplement: Supplementary file 4 — Supplementary Figure 3 [file 41398_2023_2532_MOESM4_ESM.pdf]

(A)

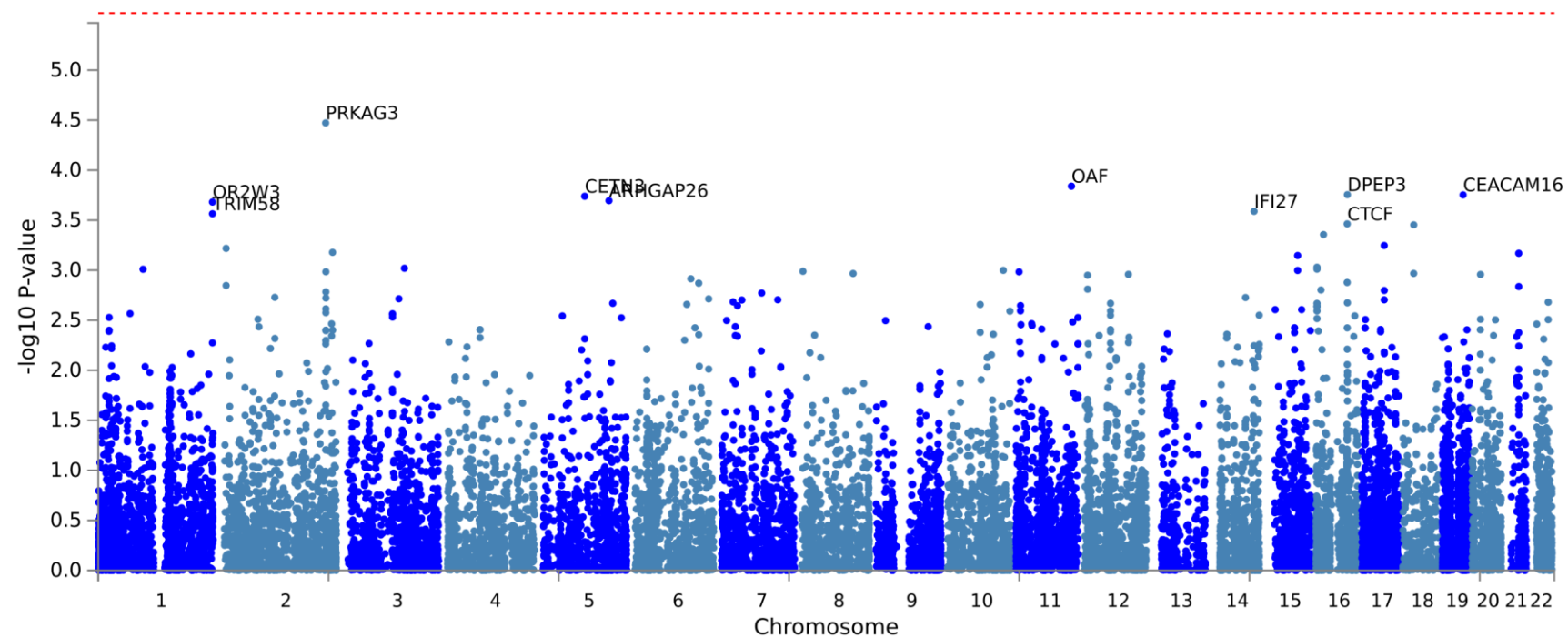

(B)

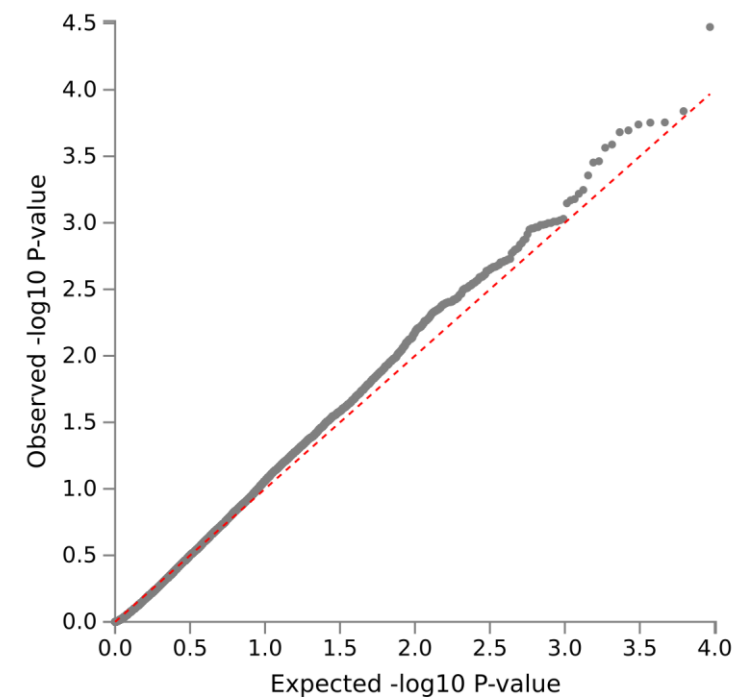

Supplement: Supplementary file 5 — Supplementary Figure 4 [file 41398_2023_2532_MOESM5_ESM.pdf]

(A)

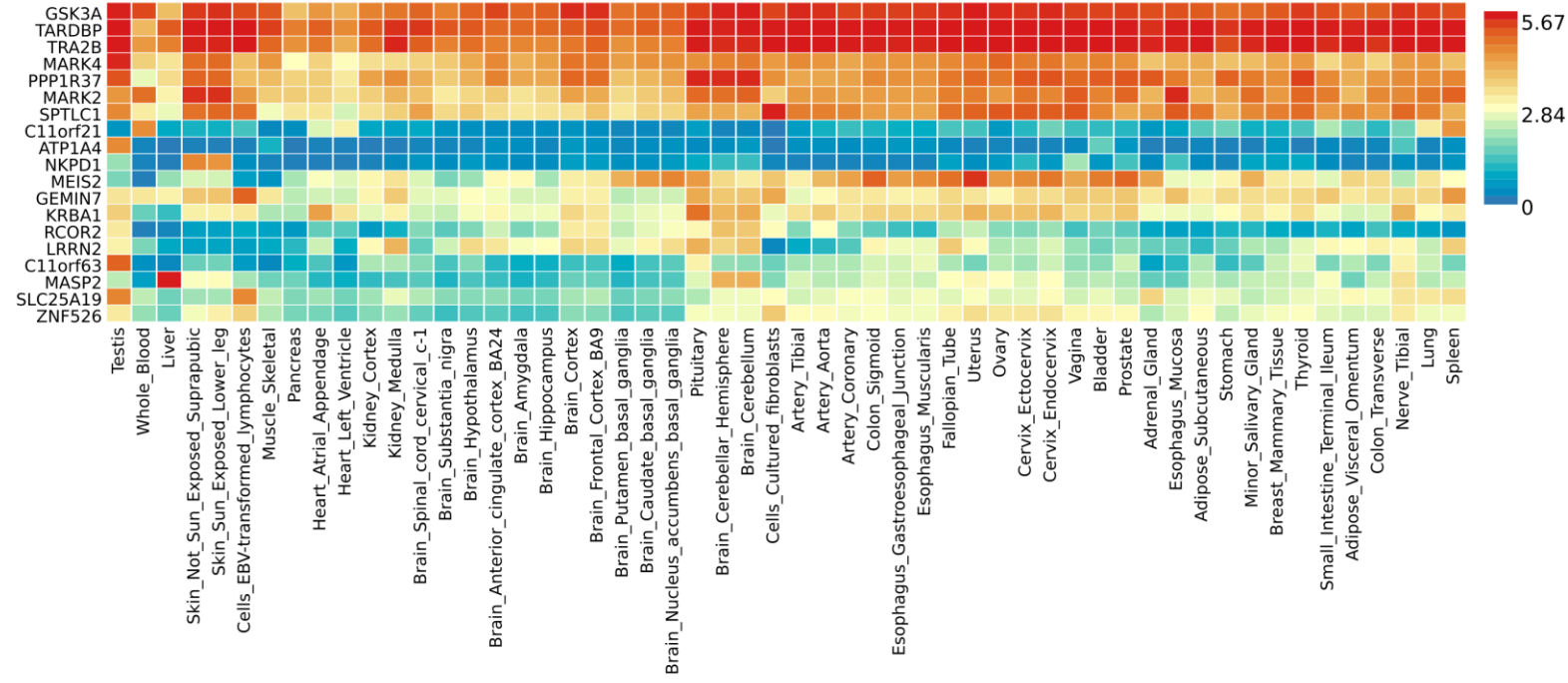

(B)

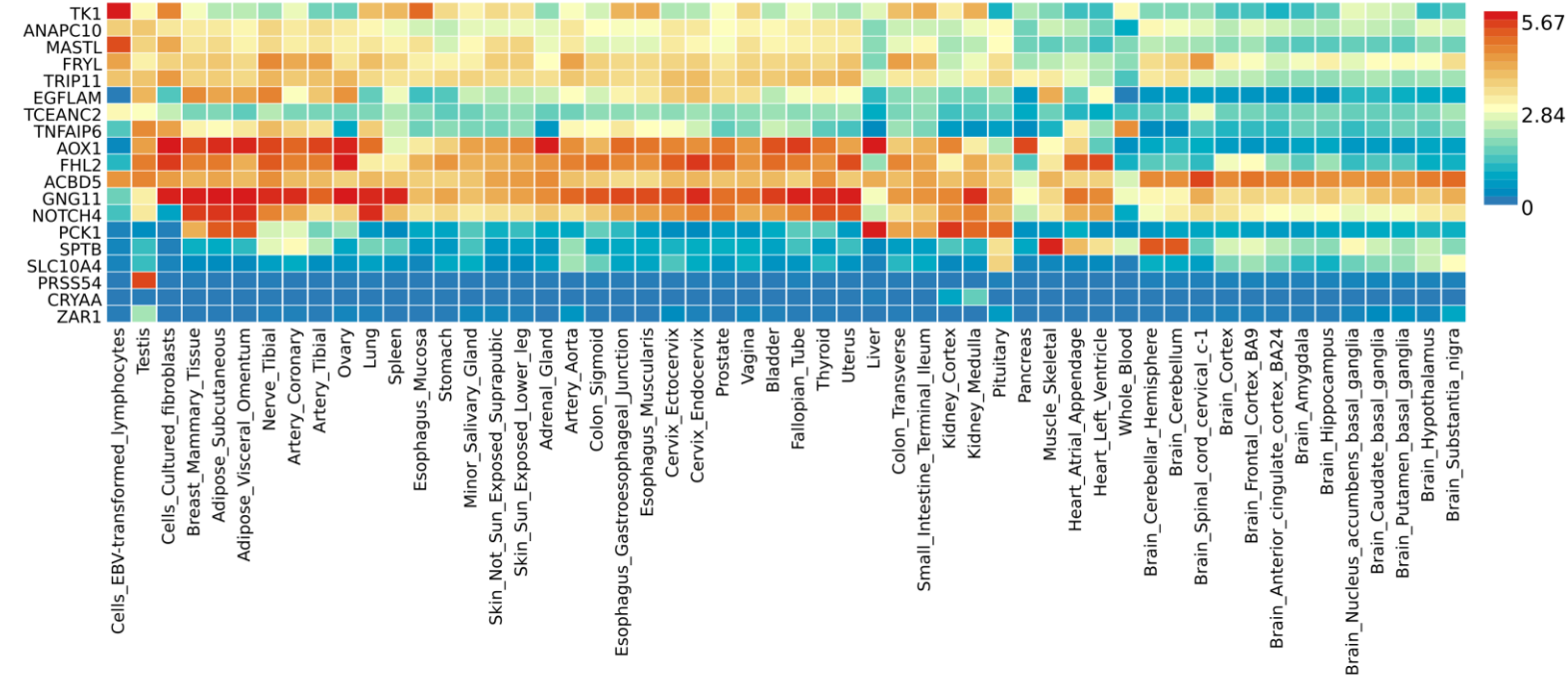

Supplement: Supplementary file 6 — Supplementary Figure 5 [file 41398_2023_2532_MOESM6_ESM.pdf]

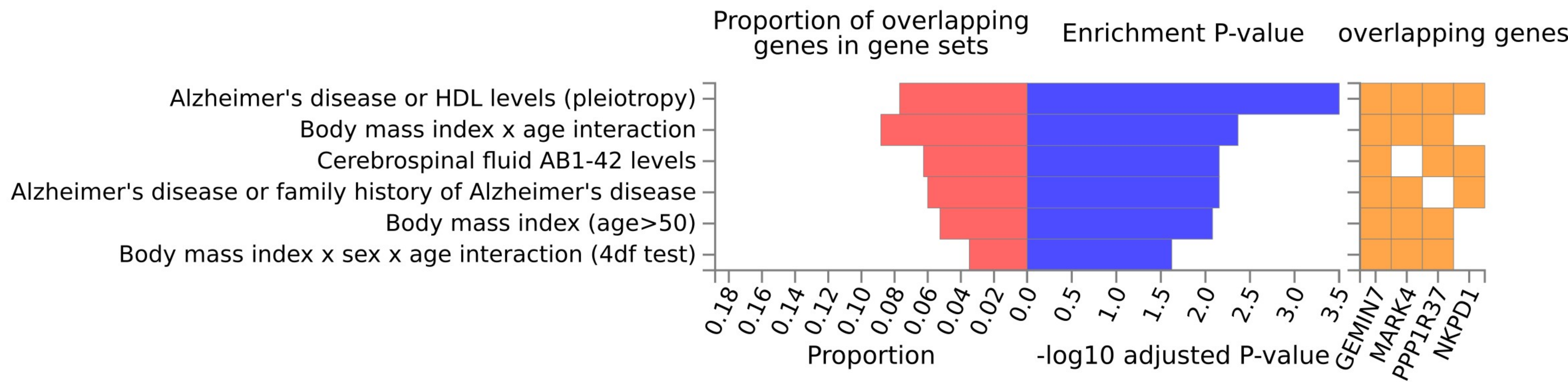

Supplement: Supplementary file 7 — Supplementary Figure 6 [file 41398_2023_2532_MOESM7_ESM.pdf]
